# Philadelphia Hospital

**Published:** 1844-12-28

**Authors:** Samuel G. White


### THE MEDICAL EXAMINER,

### And Record of Medical Science.

Vol. VII.] PHILADELPHIA, SATURDAY, DECEMBER 28, 1844. [No. 26.

### CLINICAL LECTURES AND REPORTS.

### PHILADELPHIA HOSPITAL.

CLINIC OF PROFESSOR DUNGLISON.

*November* 30, 1844.

(Reported by Mr. Samuel G. White.)

The attention of the class was first directed to the progress of the several cases that had been under consideration at the former clinics.

The patient suffering from

ULCERATION OF THE LOWER PORTION OF THE INTESTINAL CANAL, whose history was detailed in the last lecture, had experienced much benefit from the plan of treatment then directed, the evacuations being not only improved in character, but diminished in number. The treatment, as it has been thus far attended with good effects, will be continued, only increasing the strength of the injection to 5 grs. of the sulphate of copper to the ounce of water; still carefully regulating the diet.

The case of

SCARLATINA

under the simple treament before indicated, has proceeded most favourably, the eruption having wholly disappeared, and the patient being convalescent.

The female, labouring under

TYPHOID FEVER

of about three weeks duration, had likewise much improved—the pulse being as low as 60, and no unfavourable symptom present. It may be here remarked, that in the commencement of typhoid fever, the pulse is often frequent, but towards the close, it subsides, even beneath the healthy standard. The lecturer observed, that the patient at present is in that state of adynamia which generally succeeds to the period of febrile excitement in these fevers, and demands the administration of tonics. The course of treatment pursued in this case was similar to that required in ordinary simple remittent fever; the bowels were kept free from morbid secretions, cool drinks were allowed freely, and the heat of surface was tempered by ablutions with tepid or cold water; the diet being at the same time carefully regulated, and irritants of every character excluded. Under this simple mode of treatment, this case has been conducted through the various stages of the disease, and convalescence is now fairly established. Although there was gurgling in this case, in the right iliac fossa, it was unaccompanied with tenderness on pressure. With the exception, indeed, of the diarrhœa, there was no evidence in this instance of inflammation and ulceration of the glands of Peyer; and the patient having recovered, there can be no mode of knowing whether such ulceration existed.

The lecturer next introduced a patient to exhibit, principally, the beneficial effects of the topical application of the tincture of iodine in certain affections. A female had suffered severely from

ERYSIPELATOUS INFLAMMATION OF THE SCALP, accompanied by great hyperæsthesia of the integuments. Conjoined with this, there was inflammation of the cervical lymphatic ganglions, some of which had suppurated. She was manifestly of the strumous diathesis, to which the glandular affection must be referred, for although irritation of the scalp, as of any other part, might occasion enlargement of the lymphatic ganglia situate between the seat of irritation and the thoracic duct, yet in this instance, it was not dependent upon this cause, as the glands were inflamed before the scalp was affected.

After the usual means of removing the inflammation had proved unsuccessful, the lecturer directed the scalp to be penciled with the tincture of iodine, which was followed by most beneficial results, relief being obtained in a very short time. He thinks, that this agent acts in a manner similar to the nitrate of silver, that is, by forming a pellicle, which protects the surface from the irritating influence of the air, and by its excitant properties inducing, at the same time, a new action in the vessels of the inflamed surface. For the dyscrasy under which she labours, the iodide of potassium has been directed, conjoined with other means of improving the general health, as exercise in the open air, good diet, &c.

When a strumous or other *vice* obtains in the system, those remedies have to be administered, which, by being absorbed into the mass of blood, may alter the condition of the fluid, and in this manner modify the nutrition of the parts which it bathes, and it can be readily conceived that a salutary change might thus be produced in the whole system. It is by this modification of the circulating fluid, that the different panaceas which he instanced probably produced their effect.

The lecturer then proceeded to the investigation of

DISEASES OF THE RESPIRATORY ORGANS, the consideration of the physiological action of these organs, having engaged the class in the college for some days previously. The subject of the first case being too feeble to be introduced before the class, a short history of the affection and its treatment was given.

The patient labours under what has been called, with doubtful propriety,

NECROPNEUMONIA, inasmuch as it is not settled, that gangrene of the lung must necessarily result from pneumonia, but may be owing to an opposite condition; as in the form of ordinary gangrene, in which there is obliteration of the principal blood vessels of a part.

When gangrene attacks the lung, it may, in its commencement, present merely undefined evidences of inflammation of the bronchical mucous membrane, followed or accompanied by signs of consolidation of the pulmonary tissue; and finally there may be cavernous respiration, gurgling and other signs of the presence of a cavity. These signs are, however, somewhat anomalous and indistinctive, and the only one on which much reliance can be placed, is excessive fœtor of the breath, and of the matter of expectoration. But in the present instance, this sign might be considered equivocal, as the patient had shortly before, from the presence of chronic bronchitic and pneumonitic phenomena, been put upon the use of mercury so as to produce its effects upon the system. The effect of that remedy had, however, passed away, before the fœtor of gangrene presented itself.

The prognosis in gangrene of the lung is always unfavorable, and some writers of eminence have stated that death is its invariable consequence. The lecturer however, witnessed a case, during his attendance as one of the physicians to the Baltimore infirmary, in which the excessive fœtor was present, with other signs, that led to the conviction of the existence of gangrene, but the patient eventually recovered. He believes, that under peculiar circumstances, the secretion of the bronchia may assume so closely the characters of that which occurs in gangrene, as to be mistaken for it, and that it is possible, that the cases of gangrene of the lungs reported to have recovered were of this nature.

The treatment of this affection can only be palliative, for no remedy can be brought to act directly on the diseased part; the only hopes of recovery rest on the possibility of the part affected sloughing away. Usually, however, to correct the fœtor, which is harassing to the patient, and almost intolerable to the attendants, the various chlorinated preparations, especially chlorinated lime, four or five grains of which may be given several times a day, in the form of pills, are administered, but manifestly without any rational expectation of effecting a cure.

The next patient which the lecturer had intended to bring before the class, was also too ill to be introduced, and he made it a point never to incur the least risk. It was a case in which

TUBERCULAR DEPOSITS

had probably taken place, at the summit of the lungs, which had given occasion to copious hœmoptysis. It was, however, difficult to say, from the superficial examination that had been practicable, whether the hæmoptysis was dependent on softening of tubercle with ulceration of vessels, or whether it might not be a simple transudation from mechanical impediments to the circulation, produced by tubercles—as explained on a former occasion. It was more probable, however, that it occurred in the latter mode. In this instance, as the patient was very feeble, and no excitement of the circulation existed, bloodletting could not be demanded, and if employed might be injurious. The treatment had therefore consisted in the use of ice to moderate thirst when it existed, and in abstracting every thing calculated to excite the system. With the view of producing a revulsive impression on the intestinal canal, a dose of oil of turpentine with castor oil has been prescribed. The turpentine seemed peculiarly appropriate in this case, as it is esteemed by some to be one of the best hæmastatics, whilst at the same time it powerfully impresses the intestinal mucous membrane. The patient has been put, by the resident physician,—Dr. Curran—on the use of the tincture of chloride of iron, but if it does not prove beneficial it will be suspended, and the plumbi acetas combined with opium be substituted,—two or three grains of the former to from a quarter to half a grain of the latter, being given every five or six hours.

The lecturer here observed, that he was extremely desirous of impressing on the class the importance of always investigating the pathological cause of such affections and not to prescribe merely for the hemorhage. This he considered especially important in determining the propriety of practising bloodletting for the arrestation of hemorrhage, for, although under certain conditions, when there is manifest vascular excitement, the measure may be productive of much good, it may, under different circumstances, prove most injurious. The feeling, that we must bleed, because a patient is bleeding, he considered a most unfortunate one.

Another circumstance of much importance to be borne in mind in these cases, is that the rapidity of absorption is in an inverse proportion to the fulness of the vessels.—Bearing this in mind, the impropriety of permitting the free use of diluents, after copious bloodletting or hemorrhage, will be at once perceived. The fluids, under such circumstances, pass freely into the mass of blood, distend the vessels, and by thinning the blood, lay the foundation for the recurrence of the hemorrhage. It should ever be remembered that imbibition is increased by depletion; and that transudation is favored by diminishing the consistence of the blood. Magendie, has properly declared, that one of the greatest errors, prevalent among practitioners, is that of allowing the free use of drinks in cases of hemorrhage. If the patient demands something to allay thirst, let portions of ice be given, which will be most grateful, and cannot easily furnish a large amount of fluid. It will usually be found, that the hemorrhage, after a certain amount has escaped, will cease of itself, and the common plan of placing salt in the mouth, and *à fortiori* of giving a solution of the same, can exert no influence in arresting it. It cannot come in contact with the vessels that are concerned in hæmoptysis, and the lecturer cannot comprehend on what principle it has been proposed. He never prescribes it. By Andral, it is referred to as a remedy employed in Philadelphia! The lecturer urged upon the class the importance of putting a hemorrhagic patient as much as possible upon a *dry* diet.

The attention of the class was next directed to the case of Jno. McB. who was exhibited on a former occasion, with

ULCERATION OF THE THROAT

involving the larynx. The condition of the throat, which was in a lax and ulcerated state, had much improved under the treatment employed. It was remarked before, that it was very probable, that the pulmonary tissue had become implicated, and that there might be tubercular deposition. From the patient's statement, he has suffered from hoarseness and cough for more than three months, but has no pain, except a slight tenderness which he refers to the lower part of the anterior mediastinum. There are no positive signs of hectic present, although there is sometimes heat of skin, with irregular night sweats. The lecturer, then proceeded to examine the patient's chest, in the presence of the class. On applying the stethoscope along the larynx, no rhonchus was audible, but there was an unusual roughness in the right side, during inspiration. Inspection of the chest, detected a greater depression under the right clavicle than under the left; and the expansion, during inspiration, seemed diminished at the same point. When percussion was made over these regions, there was slight dulness under the right clavicle, indicating that there was some increase of density in the parts beneath; and when the ear—the lecturer always preferring the *immediate* method — was applied. the expiratory murmur appeared considerably prolonged, owing no doubt to the presence of some impediment to the free egress of the air. Combining the evidence afforded by those signs, there seems no doubt of the existence of some degree of consolidation at the summit of the right lung, which is probably tubercular.

Continuing the investigation of this class of affections, the professor next introduced a more marked case of

PULMONARY PHTHISIS.

The patient, Jno. McC., æt. 33, entered the hospital a few days since, for a cough, with copious expectoration of purulent matter. It appears, that three years previously, he had suffered an attack of pleuritis, and that the cough has continued since that period, subject to occasional exascerbations. During last winter the expectoration became purulent in character, and within the two last weeks he has complained of some pain in the chest. He never had hæmoptysis; and at present there seems to be no well marked hectic, although he is becoming more debilitated, and has occasional febrile excitement.

In this instance, then, the functional signs of phthisis, are present, but not well developed; the physical signs are more marked.

From simple inspection it was very evident, that the left side is generally fuller than the opposite, and the expansion on inspiration greater. This fulness, with the increased expansion may be explicable from the fact, that in consequence of the pleuritis the right lung has been bound down so as to be nearly useless, and has therefore become atrophied, whilst the left may have become somewhat hypertrophied from the augmented action it has been required to perform.

On percussing over the infra-clavicular regions, the difference in the resonance of the opposite sides was very obvious, that of the left being unusually clear, which may be owing to slight emphysema, whilst that of the right was quite flat. The resonance on percussion was also very great over the region of the heart, where it should be dull, owing no doubt to the presence of the fully developed lung between it and the thoracic parietes. The respiratory murmur was combined with crackling, which is indicative of an emphysematous condition of the lung. This however was very slight.

On the right side, the respiratory murmur was nearly audible, but a slight subcrepitant rhonchus was heard, caused probably by the bursting of minute mucous bubbles, contained in the smaller bronchial tubes. The vocal resonance in the left was natural, but extremely marked in the right side, and the lecturer observed, that there could be no better marked case of increased vocal resonance or bronchophony than this presented. By associating these various phenomena, derived from the physical signs, the diagnosis of consolidation and softening was rendered certain, and combining these with the functional phenomena, there could be no doubt it was a case of pulmonary consumption. The professor here exhibited the expectoration of the patient, which showed, in a marked manner, the distinction between the bronchial mucous secretion and the tubercular matter. Some reflections were indulged in on the distinctive characters presented by this case, and on the marked difference between the physical signs in tubercular consolidation, bronchitis and pneumonia. In bronchitis, there is inflammation, with thickening of the mucous membrane and increase of the secretion, but it is confined to the larger tubes, and as the matter is expectorated as it is secreted, it produces no perceptible alteration in the resonance on percussion. With the first stage of pneumonia, as there is simple hyperæmia or engorgement, producing no alteration in the sounds on percussion, tuberculosis of the lungs could not readily be confounded; but, when the pneumonia passes into the second stage, in which there is an effusion of fibrin and consequent consolidation of the pulmonary tissue, it becomes more difficult to form the diagnosis. From this, however, tuberculous deposition may be distinguished by the history of the case. When consolidation occurs in pneumonia, it is preceded by the symptoms of high vascular excitement that characterize internal inflammations in general. The professor clo ed the lecture by remarking that he would continue this subject on a future occasion, when the various signs of those different diseases, with the appropriate treatment, would be elucidated on distinct cases.

